# In vitro digestion of *custard apple* pulp: bioaccessibility of phenolic compounds, bioactive amines, and effect on antioxidant potential

**DOI:** 10.1007/s00394-026-03919-7

**Published:** 2026-02-25

**Authors:** Angélica Pereira Todescato, Pollyanna Francielli de Oliveira, Patrícia Felix Ávila, Maysa do Vale-Oliveira, Flávia Beatriz Custódio, Maria Beatriz Abreu Gloria, Bruno Martins Dala-Paula

**Affiliations:** 1 Federal University of Alfenas (UNIFAL-MG), 700 Gabriel Monteiro da Silva, Centro, Alfenas, Minas Gerais 37130-001 Brazil; 2 Federal University of Espírito Santo (UFES), Campus São Mateus, BR-101, Km 60 - Litorâneo, São Mateus, ES 29932-540 Brazil; 3 Departamento de Alimentos, Faculdade de Farmácia, Universidade Federal de Minas Gerais - UFMG, Av. Presidente Antônio Carlos, 6627, Pampulha, Belo Horizonte, MG 31270-901 Brazil; 4 Departamento de Produtos Farmacêuticos, Faculdade de Farmácia, UFMG, Belo Horizonte, Brazil; 5 Present Address: PGAli, Faculdade de Farmácia, UFBA. Av. Milton Santos, S/nº - Ondina, Salvador, BA 40170-110 Brazil

**Keywords:** *Annona squamosa* L., Antioxidant, Spermidine, DPPH, ABTS, Phenolic acids

## Abstract

**Background:**

*Annona squamosa* L. (custard apple) is a tropical fruit of the Annonaceae family whose biological effects depend on the presence and gastrointestinal bioaccessibility of bioactive compounds.

**Objective:**

To evaluate the bioaccessibility of phenolic compounds and biogenic amines and to assess the effect of in vitro digestion on the antioxidant potential of custard apple pulp (CAP) using the INFOGEST protocol.

**Methods:**

CAP was analysed for total phenolic and flavonoid contents and antioxidant potential using the 2,2-diphenyl-1-picrylhydrazyl (DPPH) and 2,2′-azino-bis(3-ethylbenzothiazoline-6-sulfonic acid) (ABTS) assays. Individual phenolic compounds and biogenic amines were identified and quantified by chromatographic methods. An *in vitro* gastrointestinal digestion (INFOGEST) model was applied to determine antioxidant potential after digestion and the bioaccessibility index (BI) of target compounds.

**Results:**

CAP contained 5.51 mg GAE/g total phenolics and 3.39 mg CE/g flavonoids, with antioxidant values of 21.94 µmol TE/g (DPPH) and 29.09 µmol TE/g (ABTS). Four phenolics were detected in undigested CAP: ferulic acid (9.36 µg/g), quercetin (4.84 µg/g), gallic acid (0.57 µg/g), and caffeic acid (0.47 µg/g). Among nine amines analysed, only putrescine (15.78 mg/kg) and spermidine (7.31 mg/kg) were present. After digestion, antioxidant potential decreased by 53 % (DPPH) and 29 % (ABTS). BI values were 58 % for total phenolics and 53 % for flavonoids; putrescine and spermidine showed BI of 67 % and 46 %, respectively. Myricetin and rutin were detected only in digested fractions.

**Conclusion:**

Custard apple pulp contains phenolic compounds, flavonoids, and the biogenic amines putrescine and spermidine that remain bioaccessible after in vitro digestion, despite reductions in antioxidant potential. The detection of myricetin and rutin only in digested fractions indicates digestion-related transformations and interactions between the digestive medium and the CAP matrix. These findings support further investigation of CAP as a source of bioactive compounds for nutraceutical, food, and pharmaceutical applications.

**Supplementary Information:**

The online version contains supplementary material available at 10.1007/s00394-026-03919-7.

## Introduction

Fruit-bearing plants play a crucial role in human nutrition and health due to their nutritional attributes and bioactive constituents, such as amino acids, protein, polyamines, carotenoids, vitamins and phenolic compounds, along with their effects on daily intake, bioaccessibility, and bioavailability [1].

*Annona squamosa* L., a tropical tree species of the Annonaceae family, is widely distributed in America and Asia, particularly in the Northeastern region of Brazil [2, 3], and its fruit is known as custard apple, sugar apple or “Conde” fruit. Custard apple pulp (CAP) is the edible portion of the fruit, characterized by a slightly granular texture, creamy yellow or white, sweet flavour, and low acidity. It is considered the sweetest of the Annona fruits [4]. This fruit has been valued due to various correlated health benefits including antimicrobial, antiviral, antidiabetic, antioxidant, antitumoral antihypertensive, and immunosuppressive [5–7]. According to the literature, several food components can promote these health benefits to human health, including phenolic compounds and bioactive amines [8, 9]. Moreover, CAP contains a complex phenolic profile, including free and conjugated (bound and esterified) phenolic compounds, which may contribute to its antioxidant potential and influence their release and bioaccessibility during gastrointestinal digestion [2].

Phenolic compounds have garnered significant interest as potential antioxidants and play a role in modulating signaling pathways, gene expression, and epigenetic modifications, particularly in relation to chronic non-communicable diseases such as metabolic syndromes, cancers, and neurodegenerative disorders [10, 11]. Additionally, phenolic compounds are prevalent in various widely consumed plant-based foods [2].

Bioactive amines are organic bases of low molecular weight, comprising both polyamines (such as spermidine, SPD; agmatine, AGM; and spermine, SPM) and biogenic amines (including putrescine, PUT; tyramine, TYM; histamine, HIS; phenylethylamine, PHM; tryptamine, TRM; cadaverine, CAD; and serotonin, SRT) [11]. The polyamines, SPD and SPM, are aliphatic amines which are reported to be essential components of all living cells [12, 13]. Due to their roles in cellular growth, normal function, proliferation, anti-inflammatory and antioxidant properties, and protection of cells and genes from damage of ionizing radiation, ultraviolet rays, toxic chemicals, and other stresses, polyamines have been even more the focus of research. In addition, polyamines may exert significant antioxidant effects that contribute to the prevention of chronic diseases, including a potential role in attenuating immunoallergic responses in the pulmonary and intestinal systems [8, 14, 15]. Some health benefits attributed to polyamines resemble those described for polyphenolic compounds. Therefore, polyamines could account for, or assist in, notable protective roles, including hepatoprotective capacity [16], antioxidant potential [15], and the mitigation of hypertension and cardiovascular disease incidence [13]. Furthermore, biogenic amines can be vasoactive, neuroactive, act as neurotransmitters, and modulate inflammatory responses [12, 13]. However, scarce information is available on the occurrence of bioactive amines in plants of the Annonacea family, with the exceptions of marolo, *Annona crassiflora* Mart. [11].

The influence of these bioactive compounds on living organisms is largely determined by their bioaccessibility, which denotes the proportion of a given compound that is liberated from the food matrix during digestion in the gastrointestinal (GI) tract and becomes available for absorption [1]. The bioaccessibility of polyphenols and amines depends not only on their release from the food matrix but also on factors such as solubility, lipophilicity, stability, enzymatic metabolism, and pH fluctuations within the GI tract [17]. Generally, polyphenols in raw fruits have low bioaccessibility, which depends on the food matrix and interactions with other food components, particularly carbohydrates, proteins and lipids [17, 18]. The hydrolysis of conjugated phenolic compounds, which may occur in subsequent sections of the digestive tract, often alter the intensity of the biological activity of formed metabolites compared to the initial compounds, such as the antioxidant potential [18]. On the other hand, aliphatic amines tend to be more soluble in aqueous medium; however, polyamines can interact with food components endowed with negative charges [19].

A previous study assessing bioactive amines during in vitro gastrointestinal digestion of marolo pulp (*Annona crassiflora* Mart.), a fruit from the same genus as *Annona squamosa* L., reported a significant decrease in putrescine and spermidine at the end of digestion [20]. The authors also noted that, under physiological conditions, intestinal microbiota may further modulate amine levels by increasing them via amino acid decarboxylation or decreasing them through microbial utilisation [20].

The use of in vitro gastrointestinal digestion serves as an efficient and cost-effective method to study, with reproducibility and speed, the structural alterations, digestibility, and liberation of food components, thereby enhancing insight into digestive phenomena [20, 21]. It is important to highlight that while the presence of a considerable content of bioactive compounds contributes significantly to their health-promoting effects, their actual bioavailability must also be taken into account [17]. Some studies have covered the potential of CAP regarding antioxidant activity correlation with secondary phytochemical compounds [3, 7], but so far there is a lack of reports exploring the bioaccessibility of amines and phenolics in CAP. Therefore, considering the scarcity of information on this fruit, this study assessed the impact of in vitro simulated digestion on the bioaccessibility of amines, phenols, and the effect on antioxidant activity presents in the CAP.

## Material and methods

### Reagent, enzymes and chemicals

Alpha-amylase (Sigma A-3176); bile salts (Sigma B-8756); pancreatin and pepsin from porcine gastric mucosa (Sigma P-3292 and P-7012, respectively) and the analytical standards from phenolic acids and flavonoids (caffeic acid – Sigma-C0625, catechin – Sigma-C1251, chlorogenic acid – Sigma-C3878, epicatechin – Sigma-03940590, ferulic acid – Supelco-PHR1791, gallic acid – Supelco-PHR2318, p-coumaric acid – Sigma-C9008, quercetin – Sigma- Q4951, and rutin – Sigma- PHL89270) and bioactive amines (spermidine trihydrochloride – Supelco-49761, agmatine sulfate – Sigma-A7127, putrescine dihydrochloride – Sigma-P7505, cadaverine dihydrochloride – Sigma-33220, histamine dihydrochloride – H0600000, serotonin hydrochloride – Supelco-14927, tryptamine hydrochloride – Sigma-193747, tyramine hydrochloride – Sigma-T2879, 2-phenylethylamine hydrochloride – Supelco-42681) were purchased from Sigma-Aldrich (St. Louis, USA), with degree of purity ≥ 95%. Water was purified in a Milli-Q system (Millipore, Bedford, MA). All the regents and solvents used in this study were of analytical grade, except those used in liquid chromatography which were chromatographic grade.

### Sample preparation

Fully mature custard apple fruits (approximately 5 kg) were acquired in the consumer market of Alfenas, MG, Brazil. The fruits were transported to the Laboratory of Experimental Nutrition at the Federal University of Alfenas (UNIFAL-MG) and stored in a polyethylene container at 4 °C for approximately 15 h prior to analysis.

The fruits were washed with distilled water, dried with paper towels, and seeds and peel were manually separated from the pulp. The CAP was immediately used for the in vitro bioaccessibility assays and for the preparation of extracts for the determination of phenolic compounds and bioactive amines. For extract preparation, CAP samples were homogenised in a food processor at 1600 rpm for 30 s (Model Ri7630, Philips Walita, São Paulo, SP, Brazil) and immediately processed for analysis, while the remaining portion was stored at − 20 °C in polyethylene bags.The CAP was analyzed for phenolic compounds (total and individual ones), antioxidant activity and bioactive amines. The pulp was also used for in vitro bioaccessibility studies, and each digested fraction (oral, oral + gastric, and oral + gastric + intestinal) analyzed for phenolic compounds and bioactive amines. The respective bioaccessibility indexes and the indexes of the digestion effect on the antioxidant potential were calculated.

### In vitro gastrointestinal digestion simulation

**Fig. 1:**
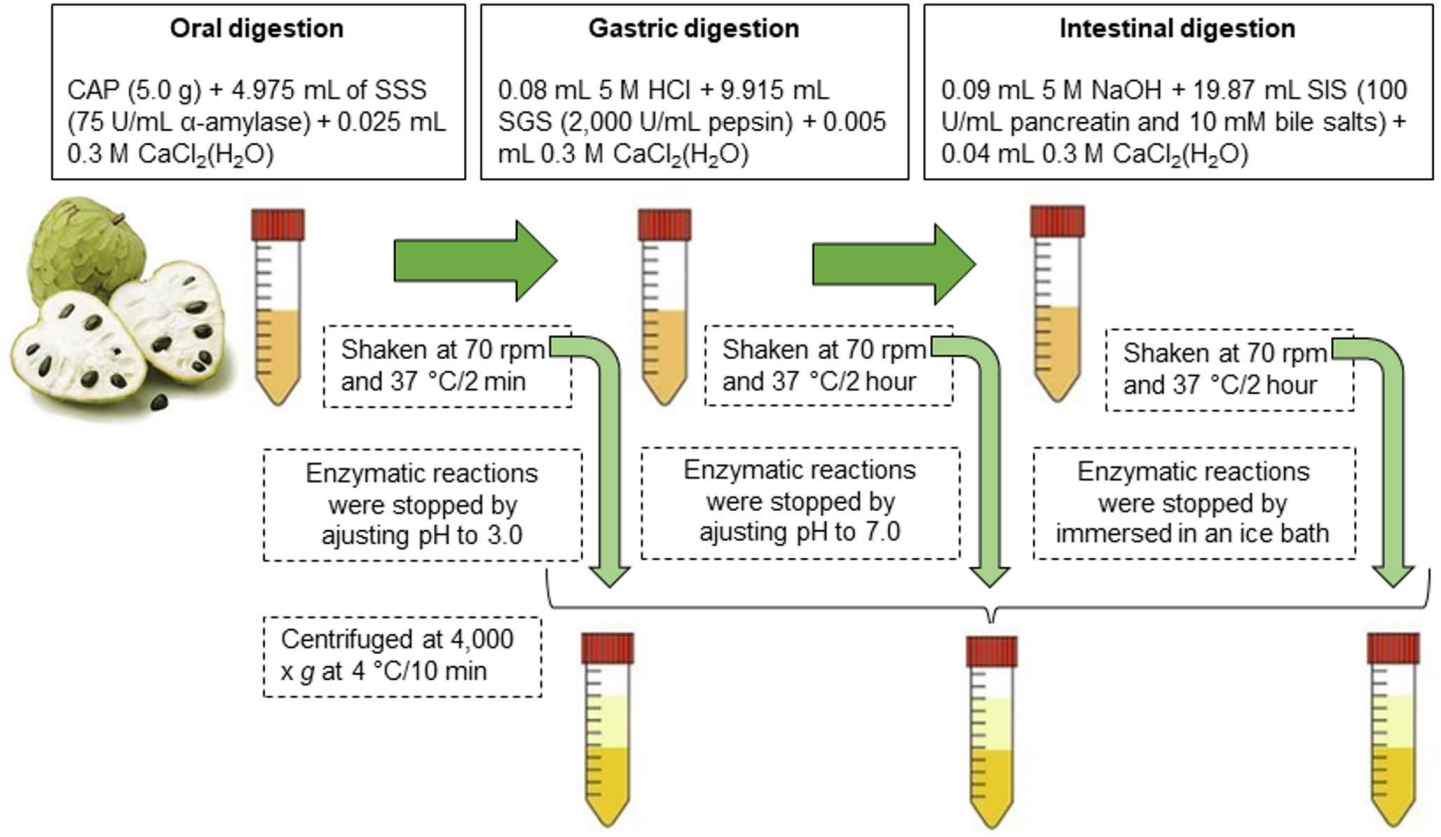
Sketch map of simulated in vitro gastrointestinal digestion of custard apple pulp (CAP)

GI digestion was conducted using the INFOGEST static in vitro method [21], incorporating minor modifications. The procedure emulated three phases of the digestive process: oral digestion alone, combined oral and gastric digestion, and the full sequence of oral, gastric, and intestinal digestion (see Fig. 1).

Digestion was performed by mixing 5.0 g of CAP, manually ground in a porcelain mortar to simulate GI digestion, starting with the oral phase, followed by the gastric and intestinal phases using three simulated fluids, simulated saliva solution (SSS), simulated gastric solution (SGS) and simulated intestinal solution (SIS), as outlined in Fig. 1. The simulated fluids were prepared as follows: SSS [15.1 mM KCl, 13.6 mM NaHCO_3,_ 3.7 mM KH_2_PO_4_, 1.1 mM HCl, 1.5 mM CaCl_2_(H_2_O)_2,_ 0.15 mM MgCl_2_(H_2_O), 0.06 mM (NH_4_)_2_CO_3_, pH 7.0], containing α-amylase (75 U/mL); SGS [47.2 mM NaCl, 25 mM NaHCO_3,_ 15.6 mM HCl, 6.9 mM KCl, 0.9 mM KH_2_PO_4_, 0.5 mM (NH_4_)_2_CO_3_, 0.15 mM CaCl_2_(H_2_O)_2_, 0.12 mM MgCl_2_(H_2_O) pH 3], containing pepsin (2,000 U/mL); SIS [85 mM NaHCO_3,_ 38.4 mM NaCl, 8.4 mM HCl, 6.8 mM KCl, 0.8 mM KH_2_PO_4_, 0.6 mM CaCl_2_(H_2_O)_2,_ 0.33 mM MgCl_2_(H_2_O), pH 7], containing pancreatin (100 U/mL), and bile salts (10 mM). Each phase mixture was agitated at 70 rpm and maintained at 37 °C for a duration of 2 h. To halt enzymatic activity prior to sampling in the oral and oral plus gastric phases, the pH was adjusted to 3.0 and 7.0, respectively. At the conclusion of the oral, gastric, and intestinal phase, enzymatic reactions were terminated by placing the test tubes in an ice bath. Subsequently, the samples from each phase were centrifuged at 4000×*g* and 4 °C for 10 min (Eppendorf SE centrifuge, Model 5810 R, Hamburg, Germany). The resulting supernatants were carefully collected, filtered, and stored in Eppendorf tubes at − 18 °C until further analysis. All procedures were performed in triplicate.

#### Bioaccessibility index (BI)

The Bioaccessibility Index (BI) of phenolics and amines in the CAP was calculated, based on the following equation:

$$BI \left(\%\right)= \left(\frac{Y}{Z}\right)\times 100$$

where Y is the bioaccessible fraction after in vitro digestion, and Z is the total content of the compound in the sample [20].

The index of the effect of digestion on the antioxidant potential was calculated based on the same formula, being named as “antioxidant potential index after digestion” (APID). In theory, bioaccessibility is applied only to nutrients or non-nutrient compounds, with antioxidant potential being a property of food, which justifies the need to adapt the nomenclature.

### Methods of analysis

#### Assessment of phenolic compounds

##### Extraction procedure

The extraction procedure to determine antioxidant activity, total phenolics, and total flavonoid content in the CAP extract was performed according to protocol described by Ávila et al. [11]. In brief, 2 g of pulp were weighed and homogenised with 10 mL of a methanol/acetone/water mixture (7:7:6, v/v/v), followed by vortex agitation for 1 min (Global Trade Technology XH-DU model, Jaboticabal, São Paulo, Brazil). The sample was then subjected to ultrasonic treatment at 25 °C for 30 min and subsequently centrifuged at 4000×g for 5 min at 4 °C. The supernatant was carefully collected. This extraction procedure was repeated twice by adding 10 mL of the same solvent mixture to the residue, followed by identical sonication and centrifugation steps. The supernatants obtained from all extractions were pooled together, and the final volume was adjusted to 30 mL using the methanol/acetone/water solution.

##### Total phenolics content

The total phenolic content was quantified using the Folin-Ciocalteu spectrophotometric method as outlined by Ávila et al. [11]. A calibration curve was constructed employing gallic acid standards at concentrations ranging from 10 to 100 µg/mL (R^2^ = 0.991). An aliquot of the appropriately diluted sample (160 µL) was combined with Folin-Ciocalteu reagent (80 µL) and sodium carbonate solution (640 µL, 75.0 g/L). Following a 30-min incubation period, absorbance was recorded at 756 nm using a spectrophotometer (BelPhotonics Ultraviolet/Visible-M51, Monza, Milan, Italy) maintained at 25 °C. Results were expressed as milligrams of gallic acid equivalents (GAE) per gram of fresh CAP (fw). The Folin–Ciocalteu assay provides an estimate of the overall reducing capacity of the extract and is not fully specific for phenolic compounds, as other reducing substances naturally present in fruit matrices (e.g., ascorbic acid) may also contribute to the measured response [22]. Therefore, total phenolic content values were interpreted as an overall index, while individual phenolic compounds were additionally assessed by UPLC-DAD.

##### Total flavonoid content

The quantification of total flavonoid content was performed following the methodology described by Ávila et al. [11]. Appropriately diluted sample aliquots (625 µL) were combined with sodium nitrite solution (37.5 µL, 5% w/v) and incubated at 25 °C for 5 min. Subsequently, aluminum chloride (37.5 µL, 10% w/v) was added and the mixture incubated under the same conditions for an additional 5 min. Afterwards, sodium hydroxide (250 µL, 1 mol/L) and distilled water (375 µL) were added consecutively, followed by agitation and incubation at 25 °C as before. Absorbance measurements were taken at 510 nm using a spectrophotometer. A calibration curve was prepared using catechin standards ranging from 4.5 to 46 µg/mL (R^2^ = 0.996), and the results were expressed as catechin equivalents (CE) in milligrams per gram of fresh CAP.

##### Assessment of phenolic compounds by UPLC-DAD

UPLC analyses targeting phenolic compounds—including gallic acid, catechin, chlorogenic acid, caffeic acid, epicatechin, ferulic acid, p-coumaric acid, rutin, and quercetin—were conducted on both the original extract (Section “Extraction procedure”) and the fractions obtained after in vitro digestion (Section “In vitro gastrointestinal digestion simulation”). These samples underwent purification via liquid–liquid extraction followed by concentration, following the procedure described by Rubio-Senent et al. [23] with minor modifications. Briefly, a 2 mL aliquot of each extract was mixed with 5.0 mL of ethyl acetate using vortex agitation. The resulting mixture was centrifuged at 1006×*g* for 10 min to facilitate phase separation and ensure the aqueous layer was fully removed. The organic phase was collected, and the aqueous phase was subjected to a second extraction with an additional 5.0 mL of ethyl acetate, followed by centrifugation under the same conditions. The combined organic extracts were then concentrated by evaporation employing a vacuum concentrator (Eppendorf, Hamburg, Germany). Finally, the dried residues were reconstituted in 2.0 mL of methanol and stored at − 18 °C until further analysis.

Chromatographic analysis was conducted following the methodology outlined by Ávila et al. [11], employing a Waters ACQUITY UPLC system (Waters Corporation, Milford, MA, USA) equipped with a quaternary pump, an online degasser, an autosampler, a column oven maintained at 35 °C, and a diode array detector. The separation process utilized an Acquity BEH C18 column (2.1 × 100 mm, 1.7 μm particle size).

For the identification and quantification of gallic acid, catechin, and chlorogenic acid, an isocratic elution was applied using 95% eluent A (water/formic acid, 99.75:0.25, v/v) and 5% eluent B (acetonitrile/formic acid, 99.75:0.25, v/v) over 8 min at a flow rate of 0.30 mL/min. The analysis of caffeic acid, epicatechin, ferulic acid, p-coumaric acid, rutin, and quercetin employed the same solvents, flow rate, and injection volume, but under a gradient elution program as follows: 0–14 min, 10–30% B; 14–16 min, 30–70% B; 16–21 min, 70–100% B; 21–22 min, 100–10% B. Quantification of the phenolic compounds was achieved by interpolation against external calibration curves (R^2^ ≥ 0.995), prepared with seven concentration levels ranging from 0.25 to 7 μg/mL, each analysed in triplicate. Detailed chromatographic parameters are provided in Table S1.

#### Assessment of the antioxidant activity

##### DPPH radical scavenging assay

The 2,2-diphenyl-1-picrylhydrazyl (DPPH) radical-scavenging activity assay was conducted following the methodology outlined by Ávila et al. [11]. In brief, 650 µL of a 0.1 mM solution of DPPH was transferred into a test tube, to which 100 µL of the sample—previously diluted according to preliminary experiments—was added. The mixture was incubated in the dark for 30 min at 25 °C before absorbance measurement at 510 nm using a spectrophotometer. A blank control was prepared for each sample, employing the respective extraction solvent or digestive fluid containing the enzymes relevant to that sample. Calibration curves were generated with TROLOX (6-hydroxy-2,5,7,8-tetramethylchroman-2-carboxylic acid) across a concentration range of 1 to 14.7 nmol/mL (R^2^ = 0.979). Antioxidant capacity results were expressed as micromoles of Trolox equivalents (TE) per gram of fresh CAP.

##### ABTS radical cation assay

The 2,2′-azino-bis(3-ethylbenzothiazoline-6-sulfonic acid (ABTS) radical cation assay was performed following the methodology described by Ávila et al. [11]. The ABTS• + was generated by combining a solution of ABTS (7 mmol/L, 5000 μL) with potassium persulfate solution (88 μL, 140 mmol/L), which was then incubated in the dark at ambient temperature for 16 h. Subsequently, the ABTS• + solution was diluted with ultrapure water to achieve an absorbance of 0.700 ± 0.005 at 734 nm, referred to as the working ABTS solution. To each assay, 750 µL of this ABTS• + solution was combined with 250 µL of the appropriately diluted sample, as determined by preliminary trials. Absorbance measurements were taken at 734 nm and 25 °C using a spectrophotometer. A blank control was also analysed, containing the extraction solvent or digestive fluid with the relevant enzymes corresponding to each sample.The analytical curve was prepared with concentrations of Trolox standard (1–17 nmol/mL) (R^2^ = 0.994). The results were expressed as µmol TE/g, fresh weight (fw).

#### Assessment of bioactive amines

Bioactive amines were isolated from approximately 5 g of CAP by performing three successive extractions using 7 mL of 5% (w/v) trichloroacetic acid (TCA). Each extraction step involved stirring for 5 min, followed by centrifugation at 11,180×*g* and 4 °C for 10 min. The resulting supernatants were then filtered through a membrane with a 0.45 µm pore size [12]. Similarly, fractions derived from in vitro digestion were filtered using the same membrane prior to analysis. The separation and quantification of nine free amines—TYM, PUT, CAD, HIS, SRT, AGM, SPD, TRM, and PHM—were conducted via high-performance liquid chromatography (HPLC) using an LC-10 AD system coupled to an RF-551 spectrofluorimetric detector. The detector was configured with excitation and emission wavelengths of 340 nm and 445 nm, respectively, and controlled by a CBM-10 AD unit (Shimadzu Corporation, Kyoto, Japan). A Luna C18 column (250 × 4.6 mm internal diameter, 5 μm particle size) was employed, adhering to the methodology previously described by Dala-Paula et al. [24]. Quantification was achieved by comparing the chromatographic peak areas to an external calibration curve prepared with standards of the nine amines across nine concentrations ranging from 0.1 to 12 µg/mL. Details regarding analytical detection parameters are provided in Table S1.

### Statistical analysis

The in vitro digestion and subsequent analytical measurements were performed in triplicate, with results expressed as mean ± standard deviation. Data analysis involved one-way analysis of variance (ANOVA), including the computation of the F statistic and its associated *p*-value. Differences between means were considered statistically significant when *p* < 0.05, and pairwise comparisons were carried out using Tukey’s test at a 5% significance threshold (Minitab® 16.2.3).

## Results and discussion

### Characterization of the custard apple pulp

The levels of total and individual phenolic compounds, the antioxidant activity and the levels of bioactive amines are described in Table 1. To the best of our knowledge this is the first study describing the contents of total phenolic content in Brazilian CAP. However, information on bioactive amines was not found in the literature, in Brazil and elsewhere. Therefore, even though custard apple fruit is very popular in Brazil, little information was available on the composition regarding bioactive compounds.

**Table 1:** Characterization of the custard apple pulp

Characteristics	Fresh pulp
Moisture	70.01 ± 0.30 g/100 g
Phenolic compounds	
Total phenolics (mg GAE/g)	5.51 ± 0.60
Total flavonoids (mg CE/g)	3.39 ± 0.22
Phenolic acids (µg/g)	
Gallic acid	0.57
Caffeic acid	0.47
Chlorogenic acid	Nd
*p*-Coumaric acid	nd
Ferulic acid	9.36 ± 0.42
Flavonoids (µg/g)	
Catechin	nd
Epicatechin	nd
Rutin	nd
Myricetin	nd
Quercetin	4.84
Antioxidant activity (µmol TE/g)	
DPPH	21.94 ± 3.30
ABTS• +	29.09 ± 3.23
Bioactive amines (mg/kg)*	
Spermidine	7.31 ± 1.42
Putrescine	15.78 ± 1.35

^*^The following amines were not detected: spermine (LOD: 0.03 mg/kg), agmatine (LOD: 0.48 mg/kg), cadaverine (LOD: 0.03 mg/kg), histamine (LOD: 0.03 mg/kg), tyramine (LOD: 0.05 mg/kg), tryptamine (LOD: 0.05 mg/kg), serotonin (LOD: 0.05 mg/kg), phenylethylamine (LOD: 0.03 mg/kg) were not detected

The mean level of total phenolic content detected in CAP was 5.51 mg GAE/g, fw. This value is akin to those documented by Ghasil et al. [25] (approximately 5.0–5.5 mg GAE/g, fw) in fruits harvested in Jhalawar, India. However, it was 0.74 times lower than reported in custard apple (7.47 mg GAE/g) from the Northern and North-Western Provinces of Sri Lanka [26]; 2.5-fold higher than custard apple (2.23 mg GAE/g,) from India [27]; and ~ 2.8-fold higher (2.07 mg GAE/g, fw) than that from Yucatan, Mexico [28]. As total phenolic content was estimated using the Folin–Ciocalteu assay, contributions from other reducing compounds cannot be excluded [22]. Nevertheless, phenolic content remains a relevant parameter for characterising fruit bioactivity due to its association with antioxidant properties [3].

The mean total flavonoid content (3.39 mg CE/g, fw) accounted for approximately 62% of the total phenolics (Table 1) and it was approximately 1.98- and 1.70-times higher than that reported in custard apple fruits harvested in Sri Lanka (1.71 mg CE/g) [26] and in Yucatan, Mexico (2.00 mg CE/g, fw) [28].

The differences observed on the levels of phenolic compounds in fruits from different parts of the world, including flavonoids, may arise from variations in species, ripeness level, cultivation region, season, harvest timing, exposure to presence of phytopathologies or to abiotic stress [29]. Considering that CAP is susceptible to enzymatic browning catalyzed by polyphenol oxidase [4], the extraction process should be carried out immediately after opening the samples, and analysis should be performed immediately.

The antioxidant potential, determined by the DPPH and ABTS methods in the CAP, was 21.94 and 29.09 µmol TE/g (fw), respectively. These results were 3.1-fold higher, and 3.6-fold higher compared to results reported to DPPH (7.03 µmol TE/g) and ABTS (8.07 µmol TE/g) methods in CAP from India, respectively [27]. The ABTS and DPPH methods determine the antioxidant potential based on the same mechanism of action, the neutralization of free radicals. However, ABTS is more sensitive to hydrophilic compounds, while DPPH is more suitable for analyzing the activity of hydrophobic compounds [30].

It is worth noting that the antioxidant potential in fruits and vegetables is related to natural antioxidant defense through different bioactive components such as vitamins, phenolic compounds, carotenoids, and polyamines [10, 11, 20]. Cell wall polysaccharides from custard apple also demonstrated potential antioxidant by DPPH and ABTS methods [31]. For example, the antioxidant activity of polysaccharides has been shown to be linked to their functional groups, including carbonyl, hydroxyl, carboxyl and amino, groups [3]. Thus, it is likely that fresh CAP contains non-phenolic antioxidants that contribute to its overall antioxidant activity. This highlights the need to identify the various constituents of the pulp using chromatographic techniques, particularly those responsible for its antioxidant effects. Therefore, the antioxidant activity of fruits can be influenced by various factors, including environmental aspects, variety, ripening, variety, antagonistic and synergistic effects [27, 31].

The types and contents of the phenolic compounds found in fresh CAP are also described in Table 1. Among the nine phenolic compounds investigated, CAP contained four of them. Ferulic acid was the predominating one (~ 10 µg/g), followed by quercetin (~ 5 µg/g), gallic acid and caffeic acid (both < 0.6 µg/g). Among the phenolic compounds investigated, Baskaran, Pullencheri, and Somasundaram [2] also detected the presence of ferulic acid, gallic acid, and caffeic acid in CAP acquired in Karnataka, India. In addition, they reported the presence of *p*-coumaric acid, catechin, epicatechin, which were not detected in this study. In addition, they reported the presence of, protocatechuic acid, epigallocatechin gallate, and sinapic acid, which were not investigated in this study. Nonetheless, quercetin was quantified in CAP and, to the best of our knowledge, it was reported for the first time in the literature. The presence of ferulic acid is interesting from the health perspective, since it offers a broad spectrum of potential therapeutic effects beneficial for cardiovascular diseases, cancer and diabetes, as well as providing neuroprotective, hepatic and photoprotective effects, along with anti-inflammatory, antioxidant and antimicrobial activities [32, 33]. In a similar way, quercetin is another interesting compound as it also exhibits anti-cancer, antibacterial, and anti-inflammatory properties [34].

Among the nine amines investigated in CAP, only PUT, and SPD were detected (Table 1), contributing 68.0% and 31.6% of the total levels of amines (23.09 mg/kg). The occurrence of SPD is of interest, as polyamines have been associated with biological functions related to cellular homeostasis and ageing in experimental models, and spermidine intake has been investigated in the context of health promotion [14, 35, 36]. However, such outcomes are generally linked to controlled intake over time [35, 36] and cannot be directly inferred from the concentrations detected in a single food matrix. In this context, the levels of putrescine and spermidine observed in CAP suggest that this fruit may contribute to dietary exposure to bioactive amines; nevertheless, the physiological relevance of these contents depends on portion size, overall dietary patterns, and inter-individual variability. Even though the role of PUT is not well established, it was reported to promote early termination of seizures in rats [37], and plays a role in gene expression regulation and intestine maturation [38].

### Bioaccessibility of phenolics compounds

The results of the bioaccessibility of total phenolics and total flavonoid content in fresh CAP are showed in Table 2. As can be seen, digestion had a similar influence on the contents of both. After oral and gastric digestion, the levels of free phenolic compounds represented approximately 21% and 26% of the levels found in the pulp before digestion, respectively. At the end of the entire process, the total phenolic compound content was 3.18 mg GAE/g, with BI of 57.7%.

**Table 2:** Total phenolics content, total flavonoids content, and antioxidant capacity (DPPH and ABTS.), in custard apple pulp (CAP) before and after in vitro gastrointestinal digestion and the respective bioaccessibility index (BI) and antioxidant potential index after digestion (APID)

Sample	Total phenolics (mg GAE/g)	Total flavonoids (mg CE/g)	DPPH (µmol TE/g)	ABTS• + (µmol TE/g)
Fresh CAP	5.51 ± 0.60^a^	3.39 ± 0.22^a^	21.94 ± 3.30^a^	29.09 ± 3.23^a^
CAP after				
Oral digestion	1.16 ± 0.13^d^	0.77 ± 0.07^d^	3.77 ± 0.35^d^	3.25 ± 0.37^d^
Gastric digestion	1.41 ± 0.24^c^	1.29 ± 0.11^c^	6.33 ± 0.95^c^	6.69 ± 1.02^c^
Intestinal digestion	3.18 ± 0.23^b^	1.81 ± 0.13^b^	10.23 ± 1.15^b^	20.62 ± 1.36^b^
BI or APID (%)	57.7	53.4	46.6	70.9

GAE: Gallic acid equivalen; CE: Catechin equivalent; DPPH: 2,2-diphenyl-1-picrylhydrazyl; ABTS^•+^: 2,2′-azino-bis (3-ethylbenzothiazoline-6-sulfonic acid); TE: Trolox equivalent. Data represents mean values ± standard deviation expressed in fresh weight (fw). The mean values with same letter in the same column did not differ at *p* < 0.05 by the Tukey’s test

This same trend was observed for the bioaccessibility of total flavonoid content, with the levels increasing throughout the GI digestion stages. After the oral and gastric stages, the total flavonoid content was approximately 23% and 38% of the level found in the fresh pulp, respectively. At the end of the simulated GI digestion, the total flavonoid content was 1.81 mg CE/g, with BI of 53.4%. Therefore, the bioaccessibility of both total phenolic content and total flavonoid content are similar, around 50% of the initial content. In fact, polyphenolic compounds in fresh fruits have low bioaccessibility [17], due to the varying degrees of solubility of phenolic compounds and their different interactions with nutrients or other components released from the food itself [9, 20]. Phenolic compounds can bind to proteins, altering their structures and functionality, and they are also unstable in acidic or even neutral pH, as exemplified by epigallocatechin-3-gallate [9, 17]. However, in a study with a fruit of the same family, araticum (*Annona marcgravii*) there was a higher BI of total phenolic content (82.6%) and of total flavonoid content (98.5%) in the fruit pulp, from 2.15 to 1.78. mg GAE/g, 4.05 to 3.99 mg CE/g, respectively [39].

The impact of in vitro digestion on the individual phenolic compounds identified in the CAP was also investigated (Table 3). Gallic acid exhibited the highest BI (107%), with is similar to what was reported in raspberry puree [40]. Caffeic acid was detected only in the CAP, below the quantification limit of the methodology used (Table S1) and was not present in any fraction of the simulated digestion. Similar to this research, caffeic acid was only detected in undigested date (*Phoenix dactylifera* L.) and was absent in all digestion fractions in two varieties of the fruit (Ajwa and Jomara dates) [41]. Ferulic acid was the main phenolic compound found in CAP and showed poor bioaccessibility, probably due to binds or interactions with carbohydrates (e.g.: pectin) and other biomolecules by etherification or esterification [31, 42], as well as its low water solubility and low stability under GI tract conditions [33]. Quercetin showed also poor bioaccessibility with a BI around ~ 37%, close to the one reported in raspberry puree (~ 33%). However, rutin and myricetin, which were not detected in the pulp, became available in the fraction after oral digestion. Rutin is an o-glycoside featuring a sugar component known as rutinoside, which is a disaccharide made up of rhamnose and glucose. It is well established that glycosidic bonds are prone to cleavage under acidic conditions and during enzymatic reactions [43].

**Table 3:** Phenolic acids and flavonoids in custard apple pulp (CAP) before and after in vitro digestion and the respective bioaccessibility index (BI)

Phenolic acids	Levels (µg/g fw)				BI (%)
	CAP	Oral digestion	Gastric digestion	Intestinal digestion	
Gallic acid	0.57 ± 0.07^ab^	0.17 ± 0.03^c^	0.46 ± 0.05^b^	0.61 ± 0.07^a^	107.0
Caffeic acid	0.47 ± 0.05	nd	nd	nd	-
Chlorogenic acid	Nd	nd	nd	nd	-
*p*-Coumaric acid	Nd	nd	nd	nd	-
Ferulic acid	9.36 ± 0.42	nd	nd	nd	-
*Flavonoids*					-
Catechin	nd	nd	nd	nd	-
Epicatechin	nd	nd	nd	nd	-
Rutin	nd	1.72 ± 0.18^a^	0.49 ± 0.04^c^	0.82 ± 0.13^b^	-
Myricetin	nd	1.07 ± 0.09^c^	1.73 ± 0.36^b^	2.71 ± 0.18^a^	-
Quercetin	4.84 ± 0.44^a^	1.13 ± 0.09^c^	1.84 ± 0.27^b^	1.78 ± 0.09^b^	36.8

nd: not detected; Data represent mean values ± standard deviations expressed in fresh weight (fw). The mean values with same letter in the same row did not differ at *p* < 0.05 by the Tukey’s test

The in vitro digestion process can also lead to the breakdown of certain phenolic compounds in highly acidic or alkaline environments [44]. According to Farias et al. [45], the reduction in the concentration of phenolic compounds after digestion may result from their sensitivity to elevated pH levels, which can render these molecules more unstable and prone to hydrolysis. Additionally, the interactions between phenolic compounds and other components of the food matrix may also influence their reduction or disappearance in the digested fraction [9, 18], which can result in the decomposition of some metabolites [44]. The changes observed in antioxidant potential throughout digestion are likely associated with structural transformations of redox-active compounds and their progressive release from the food matrix. During gastrointestinal digestion, hydrolytic conditions and enzymatic activity may promote the cleavage of esterified and glycosidic bonds, as well as partial depolymerisation or deconjugation of phenolic structures, leading to the liberation of smaller phenolic acids and flavonoids with higher measurable radical-scavenging capacity [9, 18, 45]. Therefore, the antioxidant potential detected in digested fractions may reflect not only the remaining native compounds, but also newly released or transformed phenolic constituents generated during digestion.

Similar results were documented by Giusti et al. [46], who investigated the behaviour of phenolic compounds in legumes subjected to domestic cooking and subsequent in vitro digestion. Their findings indicated that only a small proportion of phenolics was released from the legume matrix during digestion, thereby becoming available for absorption in the small intestine. More recently, it has been demonstrated that the bioaccessibility of phenolic compounds in rice bran from four Thai cultivars diminished following gastrointestinal digestion, with a notable decline in phenolic acids such as vanillic, synaptic, and protocatechuic acids, as well as flavonoids including myricetin and rutin [41]. Consequently, existing literature corroborates the results of the present study, showing that digestive simulation results in a decrease of certain phenolic constituents. Moreover, the complexity of the nutrient matrix within food may provoke synergistic, additive, or antagonistic interactions with bioactive compounds [45].

### Effect of digestion on the antioxidant activity

The antioxidant potential index after digestion (APID) was approximately 47% (DPPH) and 71% (ABTS) (Table 2). The antioxidant potential determined by the DPPH method reduced significantly after the oral stage, about—83% (from 21.94 ± 3.30 to 3.77 ± 0.35 μmol TE/g), while after the gastric digestion stage it gradually increased including after intestinal digestion stage (from 6.33 ± 0.95 to 10.23 ± 1.15 μmol/g). Similar trends were observed with the antioxidant potential determined by the ABTS scavenging method. In the other hand, the higher APID observed for ABTS, (~ 71% vs. ~ 47%) could be justified by the high prevalence of hydrophilic antioxidant compounds in CAP.

The antioxidant potential values (ABTS) reported in the literature for fruit pulps of araticum (*Annona marcgravii*), which is in the same family as custard apple, papaya (*Carica papaya*), and jackfruit (*Artocarpus heterophyllus*) after in vitro digestion were 16.48, 3.83, and 3.18 µmol TE/g, respectively [39]. These values were lower than those found in CAP after in vitro digestion in this study (20.62 ± 1.36 µmol TE/g) (Table 2). In the other hand, it is important to highlight that the simulated digestion method used in the study by Pavan, Sancho, & Pastore [39] differed from the one applied in this study, as the incubation time in the gastric digestion stage was reduced by 1 h, whereas in the present work, 2 h were employed based on the INFOGEST protocol [22]. Following the same trend observed in this study, a reduction in antioxidant potential after in vitro digestion of 12 different ecotypes of plum (*S. purpurea* L.) was also reported in the literature [47]. The antioxidant potential values (DPPH and ABTS) in the fruit pulps before digestion ranged from 17.1–18.9 μmol/g and 51.8–82.3 μmol/g, respectively. After digestion, these values ranged from 12.8–15.7 μmol/g and 18.2–29.7 μmol/g for the DPPH and ABTS methods, respectively. These values were, in most cases, higher than those obtained by CAP after the simulated digestion.

### In vitro bioaccessibility of amines

**Fig. 2:**
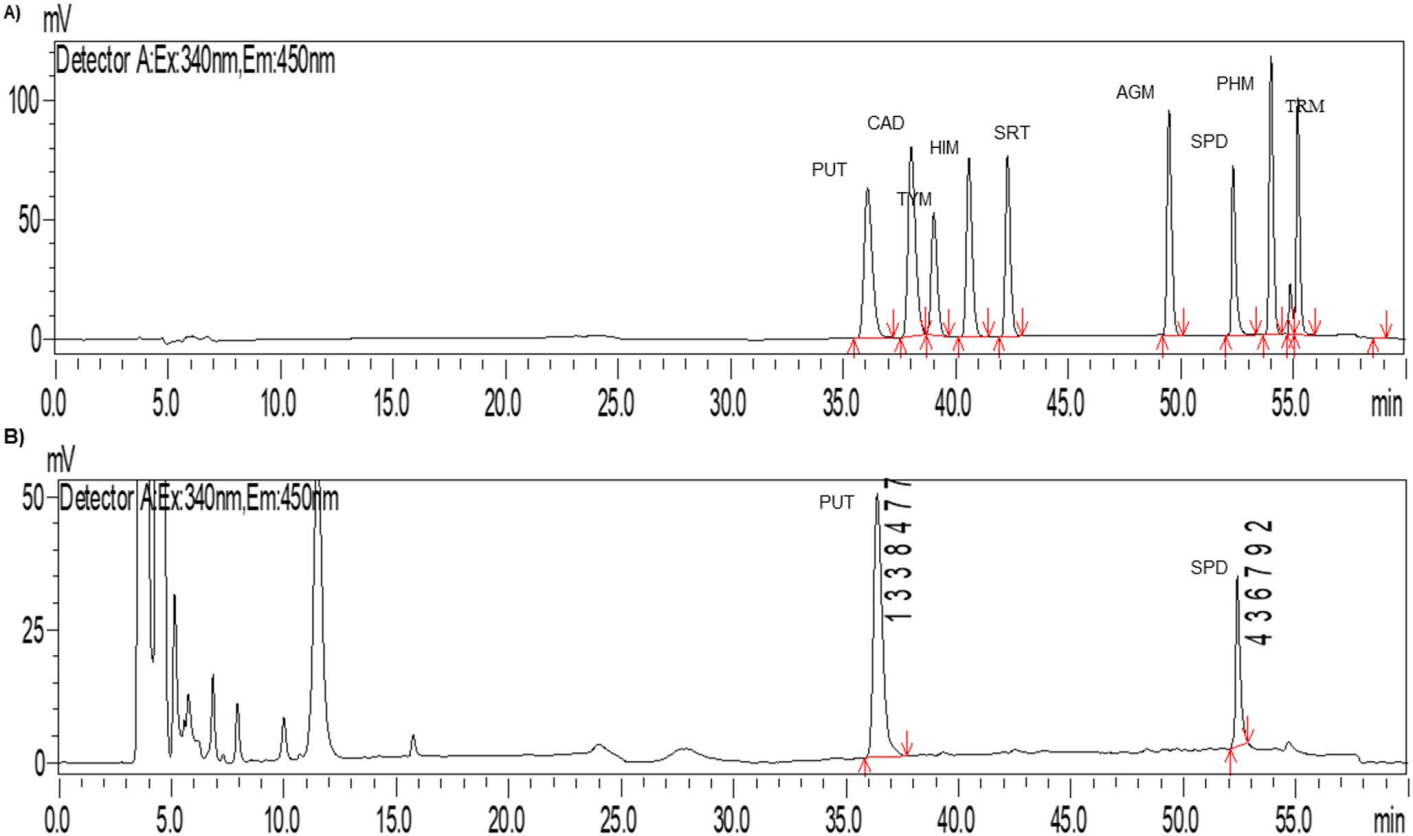
Chromatograms of bioactive amines—putrescine, cadaverine, tyramine, histamine, serotonin, agmatine, spermidine, phenylethylamine, and tryptamine— standard (A) and of custard apple pulp (B)

Among the ten bioactive amines analyzed, only putrescine (PUT) and spermidine (SPD) were present in CAP (Fig. 2 and Table 4). The content of PUT after oral digestion was around 17% of the pulp and remained unchanged after gastric digestion. However, it increased considerably after intestinal digestion, which provided a considerable BI of this diamine (66.6%). This outcome may be related to the food matrix, whereby during the digestive process of the macronutrients constituting the matrix, a gradual release of these amines occurs, similar to what is observed for phenolic compounds.

**Table 4:** Bioactive amines before and after in vitro digestion of custard apple pulp (CAP) and the respective bioaccessibility index (BI)

Extracts	Levels (mg/kg—fw)	
	PUT	SPD
Fresh CAP	15.78 ± 1.35^a^	7.31 ± 1.42^a^
CAP after		
Oral digestion	2.75 ± 0.61^c^	1.31 ± 0.28^bc^
Gastric digestion	2.91 ± 0.11^c^	0.31 ± 0.03^c^
Intestinal digestion	10.51 ± 0.58^b^	3.35 ± 0.62^b^
BI (%)	66.6	45.8

PUT, putrescine; SPD, spermidine. Mean values ± standard deviations (fresh weight—fw) with same letter in a column do not differ significantly at *p* < 0.05 by Tukey’s test

Regarding SPD, the in vitro digestion significantly reduced the content (7.31 ± 1.42 to 3.35 ± 0.62 mg/kg). However, after gastric digestion SPD content was lower than after oral and intestinal digestions (Table 3). A hypothesis to explain these results consists of the contribution of the acidic environment to the predominance of the protonated forms of SPD, which possibly contributed to its interaction/conjugation with other negatively charged compounds, such as phenolic compounds, peptides, and proteins [19, 20]. It is important to consider that in the presence of the intestinal microbiota, there could be an increase in bioactive amines due to microbial enzymes decarboxylation of free amino acid [48].

It is important to highlight that under in vivo conditions, amines can be formed by the activity of decarboxylase enzymes. These enzymes are produced by different species and strains of microorganisms of the intestinal microbiota, under free amino acids, whose content would be increased [48]. However, the bioaccessibility tests carried out did not allow to evaluate these possible occurrences. In addition, the physiological conditions of the GI tract (i.e., pH, peristaltic contraction, presence of disorders or pathologies) can significantly affect the bioaccessibility and bioavailability of nutrients and non-nutrients in food [39]. Therefore, it was suggested that future research could explore the effect of different processing of CAP on the bioaccessibility of bioactive compounds, such as PUT and SPD, clarify evidenced in this work as potential bioactive source in cold fruit pulp, since amines have already not been explored in studies involving edible parts from custard apple.

## Conclusion

In this study, it was observed that CAP presented a considerable content of total phenolic content, total flavonoid content, SPD, and PUT which presented moderate BI (> 45% and < 70%). The high values of antioxidant potential (> 20 µmol TE/g) identified in CAP was slight reduced after in vitro digestion by ABTS method (-29%) by moderate reduced considering the DPPH method (-53%).

From the in vitro digestion analysis, it was possible to observe that the most flavonoids compounds (e.g., myricetin and rutin) were detected only in digestible extracts. indicating that these compounds are more prone to degradation during the digestive process. This may be attributed to their lower chemical complexity compared to flavonoids, which were predominantly found in glycosylated forms. Although all detected amines (PUT and SPD) exhibited a significant reduction in concentrations within the digestible extracts, they remained highly bioaccessible. Lastly, our findings can enhance the understanding of the potential interactions between the digestive medium and the food matrix of CAP. Furthermore, they may support future research aimed at elucidating the possible biological effects of CAP for the development of nutraceutical extracts for use in food and pharmacological applications.

## Supplementary Information

Below is the link to the electronic supplementary material.

Supplementary Material 1

## Data Availability

The authors declare that the data supporting the findings of this study are available within the paper. Should any raw data files be needed in another format they are available from the corresponding author upon reasonable request. Source data are provided with this paper.
