# Supplementary material for: In vitro digestion of custard apple pulp: bioaccessibility of phenolic compounds, bioactive amines, and effect on antioxidant potential
Source: Eur J Nutr. 2026 Feb 25;65(2):69. doi: 10.1007/s00394-026-03919-7 (PMC12935729; doi:10.1007/s00394-026-03919-7)
Supplement: Supplementary file 1 — Supplementary Material 1 [file 394_2026_3919_MOESM1_ESM.docx]

**Table S1**. UPLC-DAD and HPLC-FL analytical parameters in the quantification of phenolics and bioactive amines, respectively

| **Class** | **Phenolics** | **RT**  **(min)** | **λ**  **(nm)** | **R^2^** | **Limits** **(µg/ml)** | |
| --- | --- | --- | --- | --- | --- | --- |
|  |  |  |  |  | **LOD** | **LOQ** |
| **Phenolic acids** | Gallic acid | 1.39 | 271 | 0.9986 | 0.11 | 0.50 |
|  | Caffeic acid | 5.97 | 320 | 0.9986 | 0.10 | 0.50 |
|  | Chlorogenic acid | 7.67 | 320 | 0.9954 | 0.17 | 0.50 |
|  | *p*-Coumaric acid | 7.87 | 320 | 0.9997 | 0.04 | 0.25 |
|  | Ferulic acid | 9.08 | 271 | 0.9963 | 0.25 | 0.50 |
| **Flavonoids** | Catechin | 6.74 | 271 | 0.9961 | 0.25 | 0.50 |
|  | Epicatechin | 6.89 | 320 | 0.995 | 0.50 | 1.67 |
|  | Rutin | 9.72 | 320 | 0.9994 | 0.14 | 0.25 |
|  | Myricetin | 12.16 | 281 | 0.9951 | 0.44 | 1.00 |
|  | Quercetin | 15.40 | 271 | 0.9974 | 0.31 | 0,50 |
|  |  |  |  |  | **Limits (mg/kg)** | |
| **Bioactive amines** | Spermidine | 52.05 | Ex 340  Em 450 | 0.998 | 0.03 | 0.08 |
|  | Agmatine | 49.11 |  | 0.998 | 0.48 | 0.16 |
|  | Putrescine | 35.79 |  | 0.998 | 0.03 | 0.08 |
|  | Cadaverine | 37.83 |  | 0.998 | 0.03 | 0.08 |
|  | Histamine | 40.09 |  | 0.997 | 0.03 | 0.08 |
|  | Tyramine | 38.93 |  | 0.999 | 0.05 | 0.14 |
|  | Serotonin | 42.03 |  | 0.993 | 0.05 | 0.16 |
|  | 2-Phenylethylamine | 53.82 |  | 0.996 | 0.03 | 0.08 |
|  | Tryptamine | 54.86 |  | 0.994 | 0.05 | 0.14 |

RT- retention time; λ- detection wavelength; R^2^- adjusted coefficient; LOD- limit of detection; LOQ- limit of quantification; Ex.: excitation; Em.: emission.
